# Incidence and risk factors for second malignancies among patients with myeloproliferative neoplasms

**DOI:** 10.1002/cam4.5666

**Published:** 2023-02-02

**Authors:** Yuhui Zhang, Yingdi Han, Guangshuai Teng, Chenxiao Du, Shan Gao, Weiping Yuan, Lei Zhang, Jie Bai

**Affiliations:** ^1^ Department of Hematology The Second Hospital of Tianjin Medical University Tianjin China; ^2^ State Key Laboratory of Experimental Hematology, Institute of Hematology & Blood Diseases Hospital, National Clinical Research Center for Blood Diseases Chinese Academy of Medical Sciences & Peking Union Medical College Tianjin China

**Keywords:** clinical characteristics, myeloproliferative neoplasms, risk factors, secondary cancer

## Abstract

**Objective:**

The clinical characteristics and survival of patients with myeloproliferative neoplasms (MPNs) with secondary cancer were analyzed to explore the possible risk factors for secondary cancer in MPN patients.

**Methods:**

The clinical characteristics of 1060 Chinese patients with MPN were retrospectively analyzed. The Kaplan–Meier method was used to analyze the survival. The Cox multivariate regression model was used to analyze the risk factors for developing secondary cancer in patients with MPNs.

**Results:**

The 1060 patients with MPN had a median follow‐up of 10 years (range 1–50) and a median age of 55 years (range 21–86), and 497 (45.2%) were male. The proportion of PV, ET, and PMF was 52.2%, 33.5%, and 14.3%, respectively. About 28.1% (298/1060) of 1060 MPN patients died. The median survival times of the PV, ET, and PMF groups were 20, 24, and 12 years, respectively (*p* < 0.0001). In age‐ and sex‐matched healthy Chinese patients, the standardized incidence ratio (SIR) value of developing secondary cancer in MPN patients was 6.41 (95% CI: 4.90–9.48). The median survival time was 14 years in the MPN with secondary cancer group. The Cox multivariate analysis showed that age ≥ 65 years (*p* < 0.0001, HR = 5.027, 95% CI [2.823, 8.952]), MF‐1 (*p* = 0.001, HR = 2.887, 95% CI [1.503, 5.545]) were risk factors for developing secondary cancer.

**Conclusions:**

The survival of MPN patients with secondary cancer was significantly worse than that of patients without secondary cancer. Compared with normal subjects, MPN patients had a 6.41‐fold increased risk of developing secondary cancer, and age ≥ 65 years and MF‐1 were risk factors for developing secondary cancer in MPN patients.

## Graphical abstract

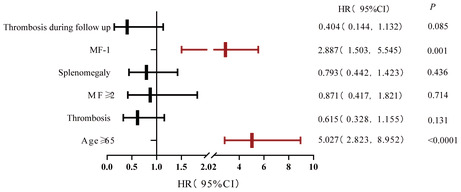

## 1 INTRODUCTION

Philadelphia chromosome‐negative myeloproliferative neoplasms (Ph‐MPNs) are malignant, clonal, proliferative diseases of hematopoietic stem/progenitor cells that includes polycythemia vera (PV), essential thrombocythemia (ET), and primary myelofibrosis (PMF). Thrombosis, secondary myelofibrosis (sMF), and secondary acute myeloid leukemia (sAML) are important complications of MPNs.^1^ In recent years, the tumors secondary to MPN has gradually attracted the attention of scholars. Studies have shown that MPN patients have a 1.5‐ to 3‐fold increased risk of developing a second cancer compared with the general population,^2^ with a 5‐year cumulative incidence of 8% and a 10‐year cumulative incidence of 12.7%.^3^ MPN patients with a second cancer had a significantly shorter survival time than MPN patients without a second cancer.^4^ Age, history of arterial thrombosis, ruxolitinib, hydroxyurea, and alkylating agents may increase the risk of second cancers in MPN patients.^5^, ^6^ However, most previous research results were derived from databases and complete clinical data is lacking. Fewer studies looked into the correlation between MPN concomitant second cancers and epigenetic‐related concomitant mutations and their risk factors, and the characteristics of second tumors in Chinese MPN patients have never been reported. Here in this study, we analyzed the occurrence and clinical characteristics of the second tumors in 1060 Chinese MPN patients, and explored the potential risk factors affecting the second cancer in Chinese MPN patients, so as to provide a theoretical basis for the prevention and rational treatment of MPN patients with second cancer.

## 2 MATERIALS AND METHODS

### 2.1 Patients

A cohort of 1060 patients diagnosed with MPNs at the Second Hospital of Tianjin Medical University and the Institute of Hematology and Blood Diseases Hospital, Chinese Academy of Medical Sciences, from January 1981 to June 2021 were selected for analysis. All MPN patients included in this retrospective study were diagnosed according to the 2016 WHO diagnostic criteria.^7^ Risk stratification for patients with PV were grouped using the 2016 WHO classification criteria, ET was grouped using IPSET, and PMF was grouped using IPSS. The diagnosis of bone marrow fibrosis after PV/ET was consistent with the International Bone Marrow Fibrosis Research and Treatment Working Group (IWG‐MRT) standard.^8^ Solid tumor patients were diagnosed according to the diagnostic criteria of the 2016 “Chinese Tumour Registration Guidance Manual”.^9^ All patients signed informed consent forms. This study was approved by the ethics committee of the Second Hospital of Tianjin Medical University.

### 2.2 Chromosome karyotype analysis

Bone marrow samples were aspirated from patients, and the nucleated cells were counted. The cells were inoculated at 1–2 × 10^6^ cells/mL in sterile bone marrow cell culture medium and cultured for 24 or 48 h in a 5% CO_2_ incubator at 37°C. The cells were treated with 0.2 μg/mL colchicine for 50 min to force the cells to remain in metaphase. The cells were collected, fixation solution was added, a cell suspension was prepared, and drops of the suspension were allowed to air dry. Finally, G or R banding was performed. After banding, chromosome image processing software was used to analyze the chromosomes of patients.^10^ At least 20 metaphase mitotic images were analyzed in each patient, and karyotypes were described according to “The International Nomenclature for Human Cytogenetics Standard” (ISCN2013).

### 2.3 Detection of *JAK2*, *CALR*, and *MPL* gene mutations

Bone marrow or peripheral blood was taken from patients, and mononuclear cells were isolated. DNA was extracted using a Small Amount Whole Blood Genomic DNA Extraction Kit (Beijing Adlai Biotechnology Co. Ltd.) and amplified with the reaction system detailed in the MPN‐related gene mutation detection kit (PCR fluorescent probe method). Driver mutations in the *JAK2* (exons 12, 14), *MPL* (exon 10), and *CALR* (exon 9) genes were examined. If the *JAK2* ^*V617F*^ mutation was detected, quantitative detection of the *JAK2* ^*V617F*^ gene mutation load was performed by StepOne real‐time quantitative PCR (Thermo Fisher Technologies). The specific detection methods were performed as previously reported.^11^, ^12^

### 2.4 Next‐generation sequencing

DNA was extracted from bone marrow or peripheral blood mononuclear cells with a Small Amount Whole Blood Genomic DNA Extraction Kit (Beijing Adlai Biotechnology Co. Ltd.) for the construction of a library with inserted fragments of 200 bp. The library was constructed using VAHTS Universal DNA Library Prep Kits (Nanjing, Vazyme). The target sequences were captured by a panel of 200 gene probes and sequenced by an Illumina Next500 second‐generation sequencing platform. To ensure the accuracy of detected variations, the average target sequencing depth of each sample was ≥2000×. The referenced human genome reference sequence is Human hg19, and NGS QC Toolkit is used for data filtering. The data were collated and quality controlled via The National Comprehensive Cancer Network (NCCN) guidelines and with dbSNP, Catalog of Somatic Mutations in Cancer (COSMIC), ClinVar, and other public and private databases, and GO functional annotation and KEGG signal pathway analysis were performed to mine gene site mutation information, determine genetic associations, and perform disease correlation analysis by using Metascape (https://metascape.org/).

### 2.5 Statistical analysis

Statistical analysis was performed using SPSS 26.0 software. Follow‐up time: time from first diagnosis to last follow‐up / time since death of patients, the last follow‐up time in this study was July 2021. For inter‐group comparison of measurement data, data conforming to a normal distribution were presented by one‐way ANOVA or T test and are represented as the mean ± standard deviation. Nonnormal data were assessed by the Kruskal–Wallis H test or Mann–Whitney U test and are presented as the median (range). Statistical data were analyzed by the chi‐square test or Fisher's exact probability method, and the test level was *α* = 0.05. If there was a statistically significant difference between the three groups, the statistic difference of two of those groups was also compared. The Bonferroni method for correction was used, and the test level was *α* = 0.05/3 ≈ 0.017. Overall survival (OS) for MPN patients was defined as the time between MPN diagnosis and death or the last follow‐up. The Kaplan–Meier method was used for survival analysis, and the log‐rank test was used to compare the survival curves of each group. The standardized incidence ratio (SIR) was calculated based on the incidence rates of age‐ and sex‐matched healthy populations. A multivariate Cox regression model was used to analyze the risk factors for developing secondary cancer.

## 3 RESULTS

### 3.1 Clinical features of patients with MPNs

The 1060 patients with MPN had a median follow‐up of 10 years (range 1–50). The median age of the 1060 patients with MPNs was 55 (21–86) years, and of these patients, 497 (45.2%) were male, with a higher proportion of women with ET (63.9%) (*p* < 0.0001). The proportion of PV, ET, and PMF was 52.2%, 33.5%, and 14.3%, respectively. Risk categorization of PV, ET, PMF was displayed in supplementary materials (Tables S1, S2, and S3). The driver mutations were *JAK2* ^*V617F*^ mutations in 84.9% (900/1060) of MPN patients, and *CALR* and *MPL* mutations in 6.2% and 1.5%, respectively. About 1.6% of patients had concomitant cytogenetic abnormalities associated with poor prognosis. Cardiovascular risk factors including hypertension, diabetes, hyperlipidemia, smoking, alcohol consumption differed among disease types (Table 1). The proportions of PV (70.3%) and PMF (78.3%) patients with splenomegaly were significantly higher than the proportion of ET (36.9%) patients with splenomegaly (*p* < 0.0001). Thrombosis occurred in 499 patients (45.4%) of which 444 (89%) had arterial thrombosis, 98 (19.6%) had venous thrombosis, and 43 (8.6%) had both arterial and venous thrombosis. Among 908 patients with PV and ET, 211 (23.2%) developed secondary MF, and the proportion of PV patients with secondary MF (26%, 144/553) was significantly higher than the proportion of ET patients with secondary MF (18.9%, 67/355) (*p* = 0.013). The incidence of sAML was 4.7% (52 cases), concomitant cytogenetic abnormalities were present at initial diagnosis in 12 (23.1%) of 52 patients with secondary leukemia, and the proportion of PMF patients with sAML (16%, 23/152) was significantly higher than that of PV patients with sAML (4.4%, 23/553) and the proportion of ET patients with sAML (1.8%, 6/355) (*p* < 0.0001). (Table 1).

**TABLE 1:** Clinical characteristics of 1060 MPN patients.

	MPN (*n* = 1060)	PV (*n* = 553)	ET (*n* = 355)	PMF (*n* = 152)	*p*	PV versus ET	PV versus PMF	ET versus MF
Median follow‐up time(range)	10 (1–50)	10 (1–50)	9 (1–33)	8 (1–28)	<0.0001	<0.0001	<0.0001	0.12
Age median (range)	55 (21–86)	55 (22–86)	53 (21–84)	57 (21–84)	0.016	0.085	0.072	0.008
Male, *n* (%)	497 (45.2%)	287 (51.9%)	128 (36.1%)	82 (53.9%)	<0.0001	<0.0001	0.654	<0.0001
Female, *n* (%)	563 (51.2%)	266 (48.1%)	227 (63.9%)	70 (46.1%)				
HB, g/L median (range)	169 (33 ~ 261)	195 (160 ~ 261)	140 (111 ~ 160)	100 (33 ~ 156)	<0.0001	<0.0001	<0.0001	<0.0001
HCT median (range)	50.35 (10.4 ~ 79.9)	59.9 (49 ~ 79.9)	42.3 (16.3 ~ 47.9)	30.65 (10.4 ~ 46.5)	<0.0001	<0.0001	<0.0001	<0.0001
WBC × 10^9^/L median (range)	11.19 (0.88 ~ 103.3)	12.5 (4 ~ 81.23)	9.7 (4 ~ 38.4)	8.94 (0.88 ~ 103.32)	<0.0001	<0.0001	<0.0001	0.157
PLT×10^9^/L median (range)	542 (9 ~ 2766)	422 (100 ~ 1972)	797 (453 ~ 2766)	227 (9 ~ 1989)	<0.0001	<0.0001	<0.0001	<0.0001
Genetic mutations								
*JAK2* ^*V617F*^	900 (84.9%)	553 (100%)	245 (69%)	102 (67.1%)	<0.0001	<0.0001	<0.0001	0.672
*CALR*	68 (6.2%)	1 (0.2%)	46 (13%)	20 (13.2%)	<0.0001	<0.0001	<0.0001	0.951
*MPL*	16 (1.5%)	4 (0.7%)	4 (1.1%)	8 (5.3%)	<0.0001	0.718	0.001	0.009
*V617F%* median (range)	43.45 (3.41 ~ 98)	51.7 (3.41 ~ 96.8)	27.9 (3.95 ~ 81.4)	50.31 (17.2 ~ 98)	<0.0001	<0.0001	0.437	<0.0001
Cytogenetic abnormalities								
Intermediate *n* (%)	1027 (98.5%)	543 (98.7%)	340 (98.6%)	144 (97.3%)	0.503	1	1	1
Poor *n* (%)	16 (1.5%)	7 (1.3%)	5 (1.4%)	4 (2.7%)				
Splenomegaly *n* (%)	639 (58.1%)	389 (70.3%)	131 (36.9%)	119 (78.3%)	<0.0001	<0.0001	0.053	<0.0001
Hypertension	433 (40.9%)	255 (46.2%)	141 (39.8%)	37 (24.3%)	<0.0001	0.064	<0.0001	0.001
Hyperlipidemia	161 (15.2%)	101 (18.3%)	48 (13.6%)	12 (7.9%)	0.001	0.066	0.002	0.074
Diabetes	140 (13.2%)	68 (12.3%)	45 (12.7%)	27 (17.8%)	0.134	0.918	0.107	0.165
Smoking	101 (9.5%)	68 (12.3%)	23 (6.5%)	10 (6.6%)	0.004	0.004	0.057	1
Alcohol	34 (3.7%)	23 (4.7%)	8 (2.6%)	3 (2.5%)	0.125	0.187	0.448	1
Thrombosis *n* (%)	499 (45.4%)	306 (55.3%)	149 (42%)	44 (28.9%)	<0.0001	<0.0001	<0.0001	0.006
Arterial thrombosis *n* (%)	444 (89%)	280 (91.5%)	128 (85.9%)	36 (81.8%)	<0.0001	<0.0001	<0.0001	0.009
Venous thrombosis *n* (%)	98 (19.6%)	58 (19%)	30 (20.1%)	10 (22.7%)	0.109	0.358	0.165	0.59
sMF *n* (%)	211 (23.2%)	144 (26%)	67 (18.9%)			0.013		
sAML *n* (%)	52 (4.7%)	23 (4.4%)	6 (1.8%)	23 (16.0%)	<0.0001	0.039	<0.0001	<0.0001
Secondary cancer *n* (%)	57 (5.37%)	29 (5.24%)	19 (5.35%)	9 (5.92%)	0.947	0.943	0.743	0.797
Death *n* (%)	298 (28.1%)	161 (29.1%)	51 (14.4%)	86 (56.6%)	<0.0001	<0.0001	<0.0001	<0.0001
Therapy								
Hydroxyurea *n* (%)	736 (69.4%)	424 (76.7%)	254 (71.5%)	58 (38.2%)	<0.0001	0.083	<0.0001	<0.0001
Ruxolitinib *n* (%)	73 (6.9%)	29 (5.2%)	15 (4.2%)	29 (19.1%)	<0.0001	0.485	<0.0001	<0.0001
Interferon α *n* (%)	626 (59.1%)	339 (61.3%)	221 (62.3%)	66 (43.4%)	<0.0001	0.774	<0.0001	<0.0001
Phlebotomy	172 (16.2%)	165 (29.8%)	7 (2.0%)	0	<0.0001	<0.0001	‐	‐
Aspirin *n* (%)	580 (54.7%)	325 (58.8%)	220 (62.0%)	35 (23%)	<0.0001	0.337	<0.0001	<0.0001

*Note*: *p* < 0.05 indicates a statistically significant difference. Abbreviations: ET, essential thrombocythemia; HCT, hematocrit; HGB, hemoglobin; MPN, myeloproliferative neoplasm; PLT, platelet count; PMF, primary myelofibrosis; PV, polycythemia vera; sAML, secondary acute myeloid leukemia; sMF, secondary myelofibrosis; *V617F%*, *JAK2* ^*V617F*^; WBC, white blood cell count.

**FIGURE 1:**
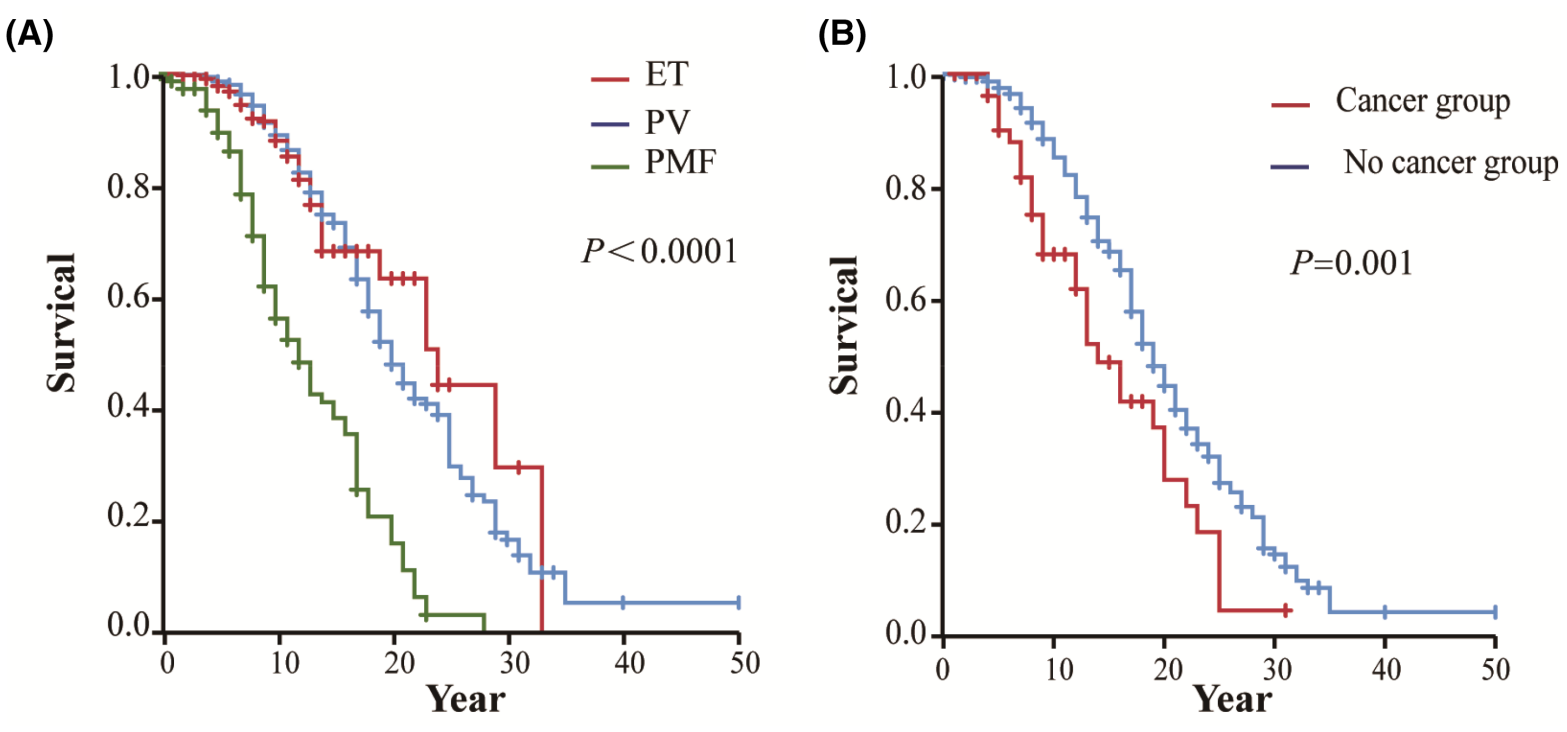
Survival analysis. (A) The survival of the PV, ET, and PMF groups (PV vs. ET *p* = 0.890; PV vs. PMF *p* < 0.0001; ET vs. PMF *p* < 0.0001). (B) Survival in the cancer and no cancer groups.

Of 1060 MPN patients diagnosed between 1981 and 2021, 28.1% (298/1060) died. The median survival time of MPN patients was 19 years, and the median survival times of PV, ET, and PMF patients were 20, 24, and 12 years, respectively (*p* < 0.0001) (Figure 1A).

### 3.2 Clinical features of MPN patients with secondary cancer

Among the 1060 patients with MPNs, 57 patients developed a secondary cancer. The incidence rates at 5, 10, and 15 years were 1.8%, 3.4%, and 4.1%, respectively. Among patients with solid tumors, there were 18 patients (1.7%) with lung cancer (the highest incidence), six patients (1.06%) with breast cancer, and five patients (0.47%) with thyroid cancer. Among the tumors of the lymphatic system, seven cases (0.66%) were lymphoma, with a relatively high incidence (Table 2).

**TABLE 2:** Clinical characteristics of MPN patients with secondary cancer.

	MPN (*n* = 57)	PV (*n* = 29)	ET (*n* = 19)	PMF (*n* = 9)	*p*	PV versus ET	PV versus MF	ET versus MF
Age median (range)	62 (23–83)	59 (40–79)	62 (23–83)	68 (43–73)	0.566	0.435	0.354	0.768
Sex								
Male, *n* (%)	32 (56.1%)	18 (62.1%)	8 (42.1%)	6 (66.7%)	0.310	0.175	1	0.418
Female, *n* (%)	25 (43.8%)	11 (37.9%)	11 (57.9%)	3 (33.3%)				
Time to secondary cancer	8 (−11–26)	13 (−3–26)	5 (−11–18)	9 (−5–21)	0.227	0.001	0.227	0.147
Secondary solid tumor *n* (%)	44 (77.2%)	23 (79.3%)	17 (89.5%)	4 (44.4%)	0.039	0.598	0.111	0.035
Lung cancer *n* (%)	18 (31.6%)	11 (37.9%)	4 (21.1%)	3 (33.3%)	0.452	0.217	1	0.815
Kidney cancer *n* (%)	2 (3.5%)	1 (3.4%)	0	1 (11.1%)	0.309	1	0.964	0.697
Stomach cancer *n* (%)	4 (7.0%)	3 (10.3%)	1 (5.3%)	0	0.398	0.929	0.766	1
Thyroid cancer *n* (%)	5 (8.7%)	2 (6.9%)	3 (15.8%)	0	0.252	0.615	1	0.554
Intestinal cancer *n* (%)	4 (7.0%)	2 (6.9%)	2 (10.5%)	0	0.444	1	1	0.822
Breast cancer *n* (%)	6 (10.5%)	2 (6.9%)	4 (21.1%)	0	0.120	0.315	1	0.364
Brain cancer *n* (%)	2 (3.5%)	0	2 (10.5)	0	0.103	0.295		0.822
Liver cancer *n* (%)	2 (3.5%)	2 (6.9%)	0	0	0.250	0.667	1	
Bladder cancer *n* (%)	1 (1.7%)	0	1 (5.3%)	0	0.327	0.830		1
Secondary haematologic tumor (not AML) *n* (%)	9 (15.7%)	4 (13.8%)	1 (5.3%)	4 (44.4%)	0.044	0.643	0.133	0.046
Lymphoma *n* (%)	7 (12.3%)	3 (10.3%)	0	4 (44.4%)	0.005	0.402	0.07	0.01
Plasma cell tumor *n* (%)	1 (1.7%)	1 (3.4%)	0	0	0.504	1	1	
Multiple myeloma *n* (%)	1 (1.7%)	0	1 (5.3%)	0	0.327	0.830		1
Type unknown *n* (%)	4 (7.0%)	2 (6.9%)	1 (5.3%)	1 (11.1%)	0.862	1	1	1
Genetic mutations								
*JAK2* ^*V617F*^	52 (91.2%)	29 (100%)	15 (78.9%)	8 (88.9%)	0.018	0.041	0.530	0.910
*CALR*	2 (3.5%)	0	2 (10.5%)	0	0.103	0.295		0.822
*MPL*	1 (1.7%)	0	0	1 (11.1%)	0.150		0.530	0.697
Therapy								
Hydroxyurea *n* (%)	40 (70.2%)	22 (75.9%)	14 (73.7%)	4 (44.4%)	0.207	1	0.174	0.278
Ruxolitinib *n* (%)	4 (7%)	2 (6.9%)	1 (5.3%)	1 (11.1%)	0.862	1	1	1
Interferon *α n* (%)	35 (61.4%)	19 (65.5%)	11 (57.9%)	5 (55.6%)	0.804	0.594	0.884	1
Aspirin *n* (%)	27 (47.4%)	13 (44.8%)	11 (57.9%)	3 (33.3%)	0.442	0.376	0.823	0.418

Abbreviations: ET, essential thrombocythemia; PMF, primary myelofibrosis; PV, polycythemia vera.

In PV patients with secondary cancer, the proportion of patients with lung cancer was the highest (37.9%, 11/29), while in ET patients, the proportion of lung cancer and breast cancer patients was 21% (4/19); and in PMF patients, the proportion of patients with lymphoma was 44.4% (4/9). The average time from ET diagnosis to secondary cancer diagnosis was 5 years, which was shorter than that in the PV and PMF groups (*p* = 0.227). (Table 2).

ET patients with secondary cancer were significantly older than those without cancer (*p* = 0.023) (Table S2). In PV patients, the incidence of thrombosis in patients with secondary cancer was 34.5% (10/29), which was lower than that in patients without secondary cancer (56.5%) (296/524) (*p* = 0.02) (Table S1). The incidence of MF‐1 in the cancer group was significantly higher than that in the cancer‐free group (MPN: *p* = 0.003, PV: *p* = 0.028, ET: *p* = 0.024). The results showed that the incidence of the secondary cancer in MPN patients with different reticular fiber grades was different. The incidence of the secondary cancer in MF‐0, MF‐1, MF‐2, and MF‐3 patients was 4%, 8.9%, 4%, and 4.7%, respectively. The incidence of the secondary cancer in MF‐1 MPN patients was the highest (Table 3).

**TABLE 3:** Differences between the MPN group with and without a second cancer.

	Cancer (*n* = 57)	No cancer (*n* = 1003)	*p*
Age median (range)	62 (23–83)	54.5 (21–86)	0.001
Male, *n* (%)	32 (56.1%)	465 (46.4%)	0.150
Female, *n* (%)	25 (43.9%)	538 (53.6%)	
HB, g/L median (range)	176 (54–242)	169 (33–261)	0.915
HCT median (range)	52.5 (21.8–74.9)	50.3 (10.4–79.9)	0.949
WBC×10^9^/L median (range)	11.34 (3–34.28)	11.19 (0.88–103.32)	0.912
PLT×10^9^/L median (range)	542 (66–1327)	542.5 (9–2766)	0.835
Cytogenetic abnormalities			
Intermediate *n* (%)	55/55 (100%)	972/988 (98.4%)	0.698
Poor *n* (%)	0	16/988 (1.6%)	
*JAK2* mutation *n* (%)	52 (91.2%)	848 (84.5%)	0.170
*V617F%* median (range)	36.15 (4.86–82.96)	43.7 (3.41–98)	0.346
Associated gene mutation (206)			
*ASXL1 n* (%)	2/17 (11.8%)	28/189 (14.8%)	1
*TET2 n* (%)	2/17 (11.8%)	32/189 (16.9%)	0.835
*DNMT3A n* (%)	2/17 (11.8%)	14/189 (7.4%)	0.865
Splenomegaly *n* (%)	32 (56.1%)	607 (60.5%)	0.511
Smoking	7 (12.3%)	94 (9.4%)	0.353
Alcohol	3 (5.2%)	31 (3.1%)	0.727
Thrombosis *n* (%)	23 (40.4%)	476 (47.5%)	0.296
Silver staining reticular fibers (1060)			
MF 0 level *n* (%)	17 (29.8%)	409 (40.8%)	0.101
MF 1 level *n* (%)	24 (42.1%)	247 (24.6%)	0.003
MF 2 level *n* (%)	7 (12.3%)	164 (16.4%)	0.416
MF 3 level *n* (%)	9 (15.8%)	183 (18.2%)	0.640
sAML *n* (%)		52 (5.2%)	0.148
Death *n* (%)	31 (54.4%)	267 (26.6%)	0.000
Therapy			
Hydroxyurea *n* (%)	40 (70.2%)	696 (69.4%)	0.901
Ruxolitinib *n* (%)	4 (7%)	69 (6.9%)	1
Interferon *α n* (%)	35 (61.4%)	591 (58.9%)	0.711
Aspirin *n* (%)	27 (47.4%)	553 (55.1%)	0.252

*Note*: *p* < 0.05 indicates a statistically significant difference. Abbreviations: HCT, hematocrit; HGB, hemoglobin; PLT, platelet; sAML, secondary acute myeloid leukemia; *V617F%*, *JAK2* ^*V617F*^; WBC, white blood cell.

The survival of MPN patients with secondary cancer was significantly worse than that of patients without secondary cancer (*p* = 0.001), especially in PV and ET patients (*p* = 0.04 and *p* < 0.0001, respectively) (Figure S1).

### 3.3 Analysis of the next‐generation sequencing results of MPN patients with secondary cancer

A total of 206 MPN patients were assessed with next‐generation sequencing, including 17 patients with MPN with secondary cancer and 189 patients with MPN without secondary cancer. Enrichment analysis of GO biological processes showed that the two groups of mutant genes were mainly enriched in the terms of histone modification, intracellular receptor signaling pathway and radiation stress response. In addition, the mutant genes in the cancer group were also involved in B‐cell differentiation. KEGG signaling pathway enrichment analysis showed that both groups had EGFR tyrosine kinase inhibitor resistance and gene mutations associated with cancer transcription disorders, but the non‐cancer group also focused on viral infection‐related pathways, and the cancer group had greater enrichment of microRNA‐related pathways and epigenetic‐related gene mutations (Figure S2A–E).

Based on the gene mutation frequency analysis, the proportion of *DNM2* mutations in MPN patients was significantly high (*p* = 0.001) (Table S4).

### 3.4 Analysis of risk factors for developing secondary cancer in MPN

**FIGURE 2:**
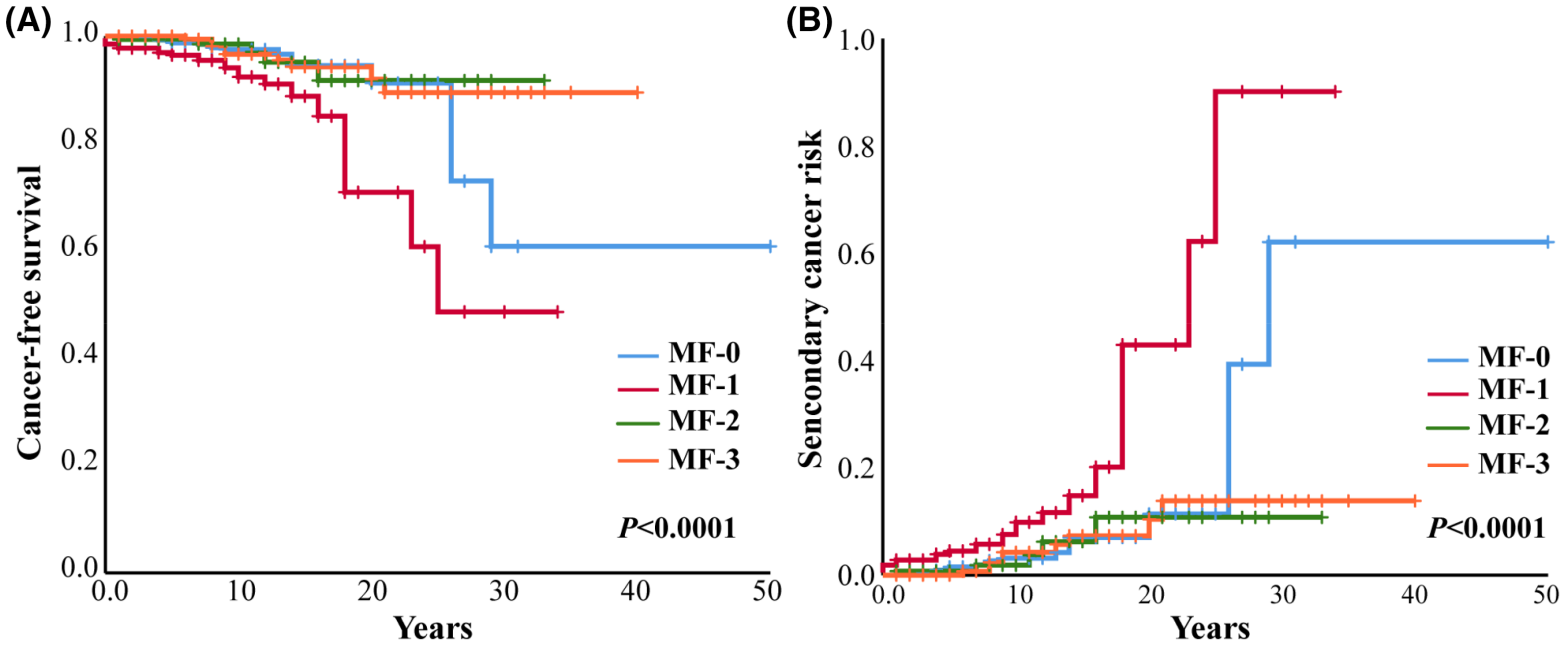
Effects of fibrosis grade on tumor‐free survival and risk of secondary cancer. (A) The tumor‐free survival of different fibrosis grade. (B) Risk of secondary cancer in patients with different fibrosis grades.

**FIGURE 3:**
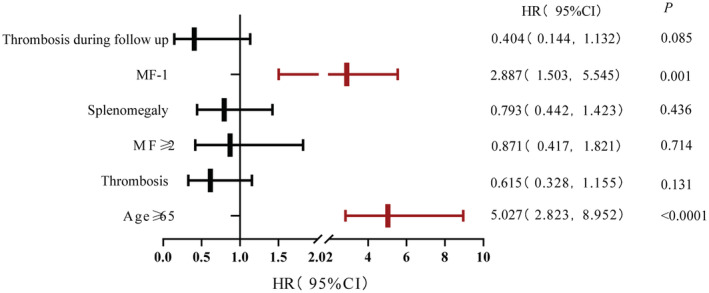
Risk factors for tumor‐free survival in MPN patients.

To establish a prognostic model to predict the risk of developing secondary cancer in MPN patients, we selected cutoff values by ROC curve and univariate analyses of continuous variables such as age at diagnosis, HCT, WBC, and PLT. Sex, spleen size, thromboembolism, secondary MF, gene mutation, chromosome abnormality, and treatment were factors included in the univariate analysis. Statistically significant factors in the Kaplan–Meier univariate analysis (such as age ≥ 65 years at diagnosis [*p* < 0.0001], MF ≥ 2 [*p* = 0.029], MF‐1 [*p* < 0.0001], splenomegaly [*p* = 0.02], thrombosis [*p* = 0.04], and thrombosis during follow‐up [*p* = 0.004] [Table S5]) were included in the multivariate analysis. The multivariate analysis showed that age ≥ 65 years (*p* < 0.0001, HR = 5.027, 95% CI [2.823, 8.952]), MF‐1 (*p* = 0.001, HR = 2.887, 95% CI [1.503, 5.545]) were risk factors for developing secondary cancer in MPN patients (Figures 2 and 3; Table S5).

In addition, we analyzed the free‐secondary cancer survival and the risk of second tumor in MPN patients with different fibrosis grades in detail. The results showed that when compared with other fibrosis grades, MF‐1 MPN patients had the worst free‐secondary cancer survival and high risk of secondary cancer (*p* < 0.0001) (Figure 2).

## 4 DISCUSSION

This study included 1060 Chinese patients with MPNs diagnosed between 1981 and 2021. The incidence of secondary cancer at 5, 10, and 15 years was 1.8%, 3.4%, and 4.1%, respectively. Compared with age‐ and sex‐matched healthy people, MPN patients in China had a 6.41‐fold increased risk from developing secondary cancer, higher than the results of European and American studies which is only 1.5‐to 3.5‐fold.^2^, ^3^, ^13^, ^14^, ^15^, ^16^, ^17^, ^18^, ^19^, ^20^, ^21^, ^22^, ^23^ Our study showed that the incidence rates of lung cancer (1.69%, 18/1060), breast cancer (1.06%, 6/563), and thyroid cancer (0.47%, 5/1060) in MPN patients were higher than those in the normal Chinese population and are different with European and American countries studies,^20^, ^24^ or South Korea.^25^ Meanwhile, we found that lung cancer (31.6%, 18/57) and lymphoma (12.3%, 7/57) were common in Chinese MPN patients, while skin cancer was rare. Interestingly, the time to develop secondary cancer is longer than observed in other studies, while the rate for developing secondary cancer is quite higher in our study. The differences in the types of secondary cancers mentioned above in MPNs in different countries may be due to the differences in geography and ethnicity. In addition, several factors may also contribute to the differences observed. For example, cancer diagnosis rates are high in European and American countries and other Asian countries, and periodic screening for cancers is well established, therefore, secondary cancers can be detected earlier. Due to the longer follow‐up period in this study, more MPN patients of advanced disease stage and advanced ages develop secondary cancer. The sample size included in this study was relatively smaller than those from European and American countries and Korea, and the result may not fully represent the incidence of secondary tumors in MPN patients in China.

Barbui found that the median time of diagnosis of secondary cancer in the PV, ET, and PMF groups was 4.6, 5.0, and 3.1 years, respectively. However, we found that the median time of secondary cancer diagnosis was 5 years in the ET, whereas it was much longer in the PV (13 years) and PMF (9 years) in our hospital. Due to the relatively smaller patient size in this study, further expansion of the sample size may be necessary to confirm this finding that the time to secondary cancer in ET patients is significantly shorter than that in PV and PMF patients.

Hansen et al.^26^ compared the incidence of secondary cancers in MPN patients treated with different treatments and showed that IFN treated patients had a significantly lower risk of developing all secondary cancers. Our study showed that the rates of IFN application in patients with MPN was 59.1%, which was significantly higher than the IFN application rate of 3% in Barbui's study. IFN can inhibit cell proliferation, regulate immunity, and have anti‐tumor effects.^27^ IFN can also change the cancer microenvironment by activating the CGAS‐STING pathway, thereby inhibiting the proliferation of cancer cells and inducing their apoptosis.^28^ We suspect that the difference in the time to diagnosis of second cancer between Chinese and Western MPN patients may be related to the high rate of IFN application in MPN patients in this study.

Next‐generation sequencing analysis has not been reported in MPN with a secondary cancer. In this study, *U2AF1*, *DNMT3A*, *SF3B1*, and *EZH2* in the secondary tumor group was higher than that in the tumor‐free group, while the mutation rate of *ASXL1*, *TET2*, and *KMT2D* was higher in the tumor‐free group. The mutation rate of *DNM2* in the cancer group was significantly higher than that in the non‐cancer group (*p* = 0.001). DNM2 mutations have been reported to be associated with poor prognosis in a variety of tumors, including acute lymphoblastic leukemia, chronic myeloid leukemia, ovarian cancer, bladder cancer, pancreatic cancer, thyroid cancer, prostate cancer, etc.^29^ In this study, the two types of solid cancers with DNM2 mutations were lung cancer and colon cancer. The GO enrichment analysis in the cancer group showed that the gene mutations were mainly enriched in the differentiation and maturation of B cells and myeloid cells. The KEGG pathway enrichment analysis found that mutations in the cancer group were mainly enriched in microRNA‐related oncogenic and epigenetic regulatory pathways. We suspected that dysregulation of intracellular signaling pathways associated with cell proliferation and enhanced ability to promote cell migration,^29^ invasion and metastasis may contribute to the second cancer.

Since age is also a risk factor for the occurrence of malignant cancers in healthy people, we include age in the risk factor analysis of secondary cancers in our MPN cohort. The results of this study showed that age > 65 years is a risk factor for secondary cancer in MPN patients. Therefore, the screening of solid tumors in older patients with MPNs should be taken as routine. Furthermore, this study found that overall thrombosis, arterial/venous thrombosis after MPN diagnosis did not increase the risk of second cancers which was different from the results of Valerio De Stefano et al.^30^ Due to the complexity of the mechanism of thrombosis, the relationship between secondary cancer and thrombosis in MPN requires further investigation.

MPN is an inflammatory disease and the expression of inflammatory factors such as IL‐6, IL‐8, TNF‐a is increased. Research shows^31^, ^32^, ^33^ that these inflammatory factors are related to bone marrow fibrosis secondary to MPN. In addition, these inflammatory factors play an important role in the development of cancer. Barbara et al. showed the incidence of SPM (second primary malignancies) was significantly higher in patients who evolved into SMF (secondary myelofibrosis) (*p* = 0.002).^34^ MF‐1 grade is found to be a risk factor for developing secondary cancer in MPN patients in this study. The incidence of the secondary cancer in MF‐1 MPN patients is 1.4 times higher than that in MF‐0 patients. This result suggests that abnormal inflammatory factors may have similar mechanisms in promoting the development of secondary bone marrow fibrosis and secondary cancers. Unfortunately, we did not find the priority of patients with MF‐ 2 and 3 MPN for concomitant secondary cancer. We speculate that it may be due to patients with MF ≥ 2 are more likely to die of organ failure and AML. We concluded that MF‐1 is correlated with the secondary cancer.

In conclusion, our study showed that the survival of MPN patients with secondary cancer was significantly worse than that of patients without secondary cancer. MPN patients have a significantly higher risk of developing secondary cancer than normal subjects, and patients with solid tumors have a shorter survival time. Lymphoma is more likely to occur in patients with PMF, and age ≥ 65 years and MF‐1 are correlated with developing secondary cancer in patients with MPNs. Therefore, attention should be given to the early screening and follow‐up of MPN patients with these risk factors. More in‐depth molecular genetics and functional studies of MPN patients will improve the understanding of secondary cancer development in MPN patients, thus provide better guidance for clinical treatment or prevention.

## AUTHOR CONTRIBUTIONS

**Yuhui Zhang:** Data curation (equal); writing – original draft (equal); writing – review and editing (equal). **Yingdi Han:** Writing – original draft (equal). **Guangshuai Teng:** Writing – review and editing (equal). **Chenxiao Du:** Writing – review and editing (equal). **shan Gao:** Visualization (equal). **Weiping Yuan:** Writing – review and editing (equal). **Lei Zhang:** Writing – review and editing (equal). **Jie Bai:** Conceptualization (lead); data curation (lead); project administration (lead); resources (lead); supervision (lead); writing – review and editing (lead).

## FUNDING INFORMATION

This work was supported in part by grants from the National Natural Science Foundation of China (82,170,117 to JB and 82,170,135 to WY).

## CONFLICT OF INTEREST STATEMENT

The authors declare no competing financial interests.

## Supporting information


Appendix S1.

## Data Availability

Data sharing is not applicable to this article as no new data were created or analyzed in this study.
