# Supplementary material for: Incidence and risk factors for second malignancies among patients with myeloproliferative neoplasms
Source: Cancer Med. 2023 Feb 2;12(8):9236–46. doi: 10.1002/cam4.5666 (PMC10166886; doi:10.1002/cam4.5666)
Supplement: Supplementary file 1 — Appendix S1. [file CAM4-12-9236-s001.doc]

**Supplementary Table 1. Comparison of clinical features between the PV cancer group and the no cancer group**

|  | **Cancer group (n=29)** | **No cancer group (n=524)** | ***P*** |
| --- | --- | --- | --- |
| **Age median (range)** | **58.86±11.75** | **54.58±12.28** | **0.067** |
| **Male, n (%)** | **18 (62.1%)** | **269 (51.3%)** | **0.260** |
| **Female, n (%)** | **11 (37.9%)** | **255 (48.7%)** |
| **HB, g/L median (range)** | **196 (170-242)** | **195 (160-261)** | **0.323** |
| **HCT median (range)** | **60.35 (52.5-74.9)** | **59.9 (49-79.9)** | **0.155** |
| **WBC, ×10^9^/L median (range)** | **16 (3.34-34.28)** | **12.46 (4-81.23)** | **0.103** |
| **PLT, ×10^9^/L median (range)** | **389 (128-1327)** | **423 (100-1972)** | **0.577** |
| **Cytogenetic abnormalities** |  |  |  |
| **Intermediate n (%)** | **28/28 (100%)** | **515/522 (98.7%)** | **1** |
| **Poor n (%)** | **0** | **7/522 (1.3%)** | **1** |
| ***VAF%* median (range)** | **48.7 (4.86-82.96)** | **51.8 (3.41-96.8)** | **0.440** |
| **Associated gene mutation** |  |  |  |
| ***ASXL1* n (%)** | **1/7 (14.3%)** | **11/93 (11.8%)** | **1** |
| ***TET2* n (%)** | **1/7 (14.3%)** | **14/93 (15.1%)** | **1** |
| ***DNMT3A* n (%)** | **1/7 (14.3%)** | **8/93 (8.6%)** | **1** |
| **Splenomegaly n (%)** | **21 (72.4%)** | **368 (70.2%)** | **0.802** |
| **Thrombosis n (%)** | **10 (34.5%)** | **296 (56.5%)** | **0.02** |
| **Silver staining reticular fibres (553)** |  |  |  |
| **MF 0 level n (%)** | **12 (41.4%)** | **247 (47.1%)** | **0.545** |
| **MF 1 level n (%)** | **13 (44.8%)** | **137 (26.1%)** | **0.028** |
| **MF 2 level n (%)** | **3 (10.3%)** | **76 (14.5%)** | **0.726** |
| **MF 3 level n (%)** | **1 (3.4%)** | **64 (12.2%)** | **0.258** |
| **sAML n (%)** |  | **23 (4.4%)** | **0.5** |
| **Death n (%)** | **18 (62.1%)** | **143 (27.3%)** | **0.000** |
| **Risk Group** |  |  | **0.156** |
| **Low** | **13(44.8%)** | **166(31.7%)** |  |
| **High** | **16(55.2%)** | **358(68.3%)** |  |
| **Therapy** |  |  |  |
| **Hydroxyurea n (%)** | **22 (75.9%)** | **402 (76.7%)** | **0.916** |
| **Ruxolitinib n (%)** | **2 (6.9%)** | **27 (5.2%)** | **1** |
| **Interferon α n (%)** | **19 (65.5%)** | **320 (61.1%)** | **0.632** |
| **Aspirin n (%)** | **13 (44.8%)** | **312 (59.5%)** | **0.117** |

**Abbreviations: HGB, haemoglobin; HCT, haematocrit; WBC, white blood cell; PLT, platelet; sAML, secondary acute myeloid leukaemia.** ***V617F%, JAK2^V617F^*.**

***P*<0.05 indicates a statistically significant difference.**

**Supplementary Table 2. Comparison of clinical features between the ET cancer group and the no cancer group**

|  | **Cancer group (n=19)** | **No cancer group (n=336)** | ***P*** |
| --- | --- | --- | --- |
| **Age median (range)** | **62 (23-83)** | **53 (21-84)** | **0.023** |
| **Male, n (%)** | **8 (42.1%)** | **120 (35.7%)** | **0.572** |
| **Female, n (%)** | **11 (57.9%)** | **216 (64.3%)** |
| **HB, g/L median (range)** | **138 (116-159)** | **140 (111-160)** | **0.295** |
| **HCT median (range)** | **40 (29.5-47.4)** | **42.5 (16.3-47.9)** | **0.194** |
| **WBC, ×10^9^/L median (range)** | **9.8 (6.03-20.2)** | **9.7 (4-38.4)** | **0.924** |
| **PLT, ×10^9^/L median (range)** | **776 (513-1193)** | **798 (453-2766)** | **0.637** |
| **Cytogenetic abnormalities** |  |  |  |
| **Intermediate n (%)** | **18/18 (100%)** | **322/327 (98.5%)** | **1** |
| **Poor n (%)** | **0** | **5/327 (1.5%)** | **1** |
| ***JAK2* mutation n (%)** | **15 (78.9%)** | **230 (68.5%)** | **0.336** |
| ***VAF%* median (range)** | **29.41 (20-58.4)** | **26.75 (3.95-81.4)** | **0.426** |
| **Associated gene mutation** |  |  |  |
| ***ASXL1* n (%)** | **0** | **6/58 (10.3%)** | **0.927** |
| ***TET2* n (%)** | **0** | **12/58 (20.7%)** | **0.492** |
| ***DNMT3A* n (%)** | **0** | **5/58 (8.6%)** | **1** |
| **Splenomegaly n (%)** | **5 (26.3%)** | **126 (37.5%)** | **0.326** |
| **Thrombosis n (%)** | **11 (57.9%)** | **138 (41.1%)** | **0.148** |
| **Silver staining reticular fibres (355)** |  |  |  |
| **MF 0 level n (%)** | **5 (26.3%)** | **162 (48.2%)** | **0.063** |
| **MF 1 level n (%)** | **11 (57.9%)** | **110 (32.7%)** | **0.024** |
| **MF 2 level n (%)** | **1 (5.3%)** | **53 (15.8%)** | **0.361** |
| **MF 3 level n (%)** | **2 (10.5%)** | **11 (3.3%)** | **0.313** |
| **sAML n (%)** |  | **6 (1.8%)** | **1** |
| **Death n (%)** | **8 (42.1%)** | **43 (12.8%)** | **0.001** |
| **Risk Group** |  |  | **0.04** |
| **Low** | **3(15.8%)** | **111(33%)** |  |
| **Intermediate** | **6(31.6%)** | **139(41.4%)** |  |
| **High** | **10(52.6%)** | **86(25.6%)** |  |
| **Therapy** |  |  |  |
| **Hydroxyurea n (%)** | **14 (73.7%)** | **240 (71.4%)** | **0.832** |
| **Ruxolitinib n (%)** | **1 (5.3%)** | **14 (4.2%)** | **1** |
| **Interferon α n (%)** | **11 (57.9%)** | **210 (62.5%)** | **0.687** |
| **Aspirin n (%)** | **11 (57.9%)** | **209 (62.2%)** | **0.707** |

**Abbreviations: HGB, haemoglobin; HCT, haematocrit; WBC, white blood cell; PLT, platelet;** **sAML, secondary acute myeloid leukaemia. *V617F%, JAK2^V617F^*.**

***P*<0.05 indicates a statistically significant difference.**

**Supplementary Table 3. Comparison of clinical features between the PMF cancer group and the no cancer group**

|  | **Cancer group (n=9)** | **No cancer group (n=143)** | ***P*** |
| --- | --- | --- | --- |
| **Age median (range)** | **68 (43-73)** | **56 (21-84)** | **0.094** |
| **Male, n (%)** | **6 (66.7%)** | **76 (53.1%)** | **0.657** |
| **Female, n (%)** | **3 (33.3%)** | **67 (46.9%)** |
| **HB, g/L median (range)** | **88.88±24.87** | **100.12±28.56** | **0.279** |
| **HCT median (range)** | **28.5 (21.8-39.4)** | **30.7 (10.4-46.5)** | **0.830** |
| **WBC, ×10^9^/L median (range)** | **7.88 (3-15.1)** | **9.3 (0.88-103.32)** | **0.173** |
| **PLT, ×10^9^/L median (range)** | **270 (66-799)** | **216 (9-1989)** | **0.553** |
| **Cytogenetic abnormalities** |  |  |  |
| **Intermediate n (%)** | **9/9 (100%)** | **135/139 (97.1%)** | **1** |
| **Poor n (%)** | **0** | **4/139 (2.9%)** | **1** |
| ***JAK2* mutation n (%)** | **8 (88.9%)** | **94 (65.7%)** | **0.285** |
| ***VAF%* median (range)** | **37.17 (30.9-43.45)** | **55 (17.2-98)** | **0.317** |
| **Associated gene mutation** |  |  |  |
| ***ASXL1* n (%)** | **1/4 (25%)** | **11/38 (28.9%)** | **1** |
| ***TET2* n (%)** | **1/4 (25%)** | **6/38 (15.8%)** | **1** |
| ***DNMT3A* n (%)** | **1/4 (25%)** | **1/38 (2.6%)** | **0.445** |
| **Splenomegaly n (%)** | **6 (66.7%)** | **113 (79%)** | **0.649** |
| **Thrombosis n (%)** | **2 (22.2%)** | **42 (29.4%)** | **0.936** |
| **sAML n (%)** |  | **23 (16.1%)** | **0.409** |
| **Death n (%)** | **5 (55.6%)** | **81 (56.6%)** | **1** |
| **Risk Group** |  |  | **0.8** |
| **Low** | **0** | **5（3.5%）** |  |
| **Intermediate-1** | **2（22.2%）** | **44（30.8%）** |  |
| **Intermediate-2** | **5（55.6%）** | **72（50.3%）** |  |
| **High** | **2（22.2%）** | **22（15.4%）** |  |
| **Therapy** |  |  |  |
| **Hydroxyurea n (%)** | **4 (44.4%)** | **54 (37.8%)** | **0.963** |
| **Ruxolitinib n (%)** | **1 (11.1%)** | **28 (19.6%)** | **0.849** |
| **Interferon α n (%)** | **5 (55.6%)** | **61 (42.7%)** | **0.681** |
| **Aspirin n (%)** | **3 (33.3%)** | **32 (22.4%)** | **0.727** |

**Abbreviations: HGB, haemoglobin; HCT, haematocrit; WBC, white blood cell; PLT, platelet;** **sAML, secondary acute myeloid leukaemia. *V617F%, JAK2^V617F^*.**

***P*<0.05 The difference was statistically significant.**

**Supplementary Table 4. Frequency analysis of concomitant gene mutations in the cancer group and no cancer group**

|  | **Cancer group (n=16)** | **No cancer group (n=200)** | ***P*** |
| --- | --- | --- | --- |
| ***KMT2D* n (%)** | **1/17 (5.9%)** | **22/189 (11.6%)** | **0.749** |
| ***ASXL1* n (%)** | **2/17 (11.8%)** | **28/189 (14.8%)** | **1** |
| ***TET2* n (%)** | **2/17 (11.8%)** | **32/189 (16.9%)** | **0.835** |
| ***DNMT3A* n (%)** | **2/17 (11.8%)** | **14/189 (7.4%)** | **0.865** |
| ***U2AF1* n (%)** | **2/17 (11.8%)** | **8/189 (4.8%)** | **0.505** |
| ***KMT2D* n (%)** | **1/16 (5.9%)** | **22/189 (11.6%)** | **0.749** |
| ***NF1* n (%)** | **1/17 (5.9%)** | **8/189 (4.2%)** | **1** |
| ***IDH1* n (%)** | **0** | **10/189 (5.3%)** | **0.702** |
| ***EP300* n (%)** | **1/17 (5.9%)** | **16/189 (8.5%)** | **1** |
| ***SETBP1* n (%)** | **1/17 (5.9%)** | **7/189 (3.7%)** | **1** |
| ***SF3B1* n (%)** | **1/17 (5.9%)** | **4/189 (2.1%)** | **0.886** |
| ***EZH2* n (%)** | **1/17 (5.9%)** | **2/189 (1.1%)** | **0.594** |
| ***SRSF2* n (%)** | **0** | **6/189 (3.2%)** | **1** |
| ***RUNX1* n (%)** | **0** | **6/189 (3.2%)** | **1** |
| ***ASXL2* n (%)** | **0** | **4/189 (2.1%)** | **1** |
| ***TP53* n (%)** | **0** | **4/189 (2.1%)** | **1** |
| ***KRAS* n (%)** | **0** | **2/189 (1.1%)** | **1** |
| ***DNM2* n (%)** | **2/17 (11.8%)** | **0** | **0.001** |
| ***NRAS* n (%)** | **0** | **1/189 (0.5%)** | **1** |

**Supplementary Table 5. Risk factor analysis**

|  | **Univariate analysis** | | | **Multivariate analysis** | | | |
| --- | --- | --- | --- | --- | --- | --- | --- |
|  | | **X** | ***P*** | | **HR** | **95% CI** | ***P*** |
| **Age ≥65 years** | | **37.115** | **0.000** | | **5.027** | **2.823, 8.952** | **<0.0001** |
| **MF≥2** | | **4.763** | **0.029** | | **0.871** | **0.****417, 1.821** | **0.714** |
| **MF-1** | | **17.402** | **<0.0001** | | **2.887** | **1.503,5.545** | **0.001** |
| **Splenomegaly** | | **5.38** | **0.02** | | **0.793** | **0.4****42, 1.423** | **0.436** |
| **Thrombosis** | | **4.238** | **0.04** | | **0.615** | **0.****328, 1.155** | **0.131** |
| **Thrombosis during follow up** | | **8.487** | **0.004** | | **0.404** | **0.144,1.132** | **0.085** |

**Abbreviations: HR, hazard ratio; CI, confidence interval.**

**P<0.05 indicates a statistically significant difference.**

**Supplementary Table 6. Incidence of secondary cancer in MPN**

|  | **MPN(n=57)** | **PV (n=29)** | **ET (n=19)** | **PMF (n=9)** | **South Korean**  **MPN(n=626)** | **South Korean**  **PV (n=387)** | **South Korean**  **ET (n=190)** | **South Korean**  **PMF (n=49)** |
| --- | --- | --- | --- | --- | --- | --- | --- | --- |
| **Lung cancer (%)** | **31.6%** | **37.9%** | **21.1%** | **33.3%** | **14.4%** | **14.2%** | **14.05%** | **18.4%** |
| **Kidney cancer (%)** | **3.5%** | **3.4%** | **0** | **11.1%** | **2.7%** | **3.2%** | **2.3%** | **4.1%** |
| **Stomach** **cancer (%)** | **7.0%** | **10.3%** | **5.3%** | **0** | **12.5%** | **10.5%** | **14.7%** |  |
| **Thyroid cancer (%)** | **8.7%** | **6.9%** | **15.8%** | **0** | **9.4%** | **8.9%** | **10.3%** | **4.1%** |
| **Intestinal cancer (%)** | **7.0%** | **6.9%** | **10.5%** | **0** |  |  |  |  |
| **Breast cancer (%)** | **10.5%** | **6.9%** | **21.1%** | **0** | **4.2%** |  | **5.7%** | **4.1%** |
| **Brain cancer (%)** | **3.5%** | **0** | **10.5%** | **0** |  |  |  |  |
| **Liver cancer (%)** | **3.5%** | **6.9%** | **0** | **0** | **9.7%** | **12.6%** | **6.5%** | **24.5%** |
| **Bladder cancer (%)** | **1.7%** | **0** | **5.3%** | **0** | **2.6%** | **3.7%** |  |  |
| **Median survival of secondary cancer patients(years)** | **14** | **19** | **12** | **13** |  |  |  |  |

**Abbreviations: PV, polycythemia vera; ET, essential thrombocythemia; PMF, primary myelofibrosis.**


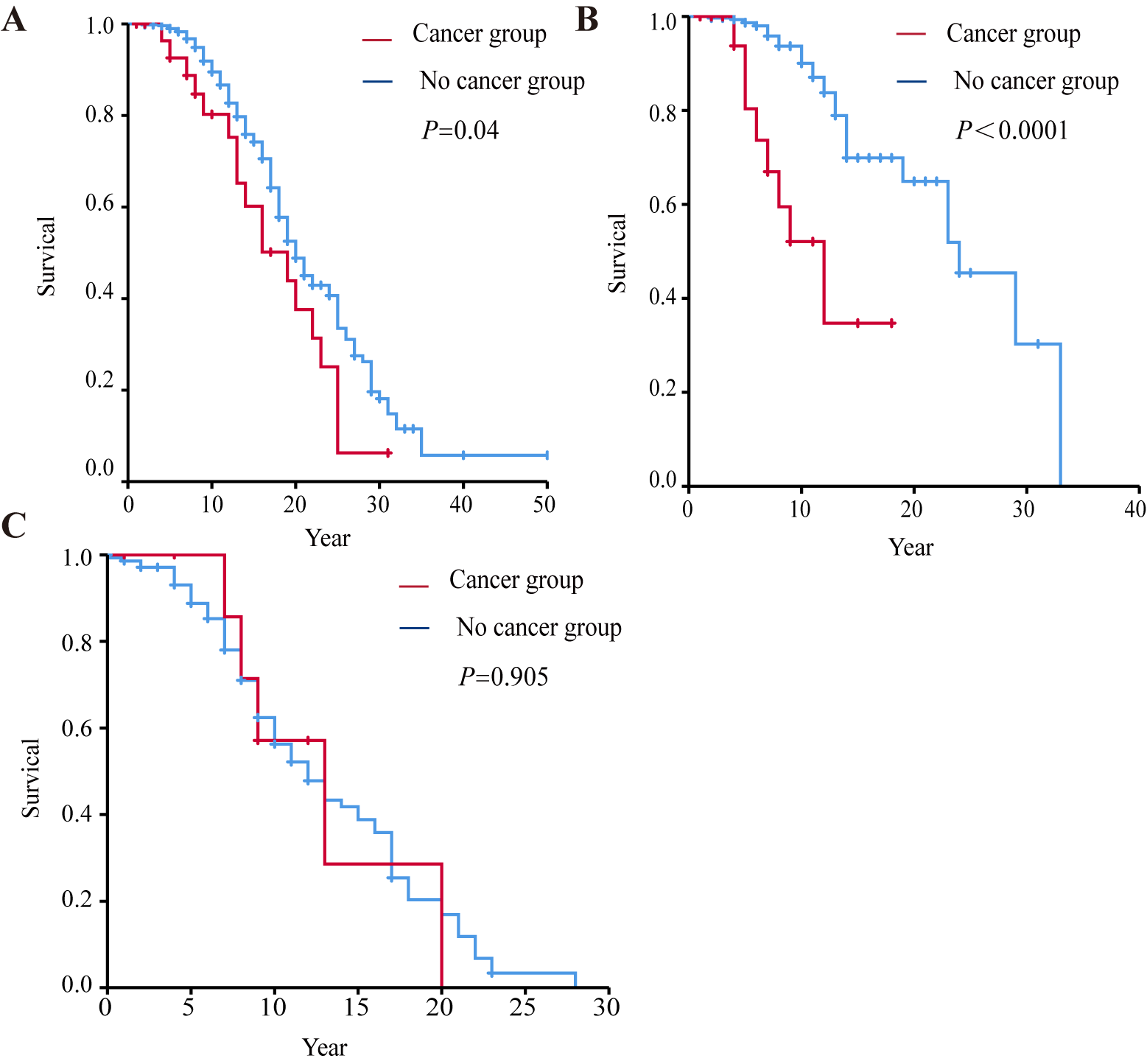
**Supplementary Figure 1. Survival analysis.** **A, PV: survival in the cancer and no cancer groups. B, ET: survival in the cancer and no cancer groups. C, PMF: survival in the cancer and no cancer groups.**

**A**

**
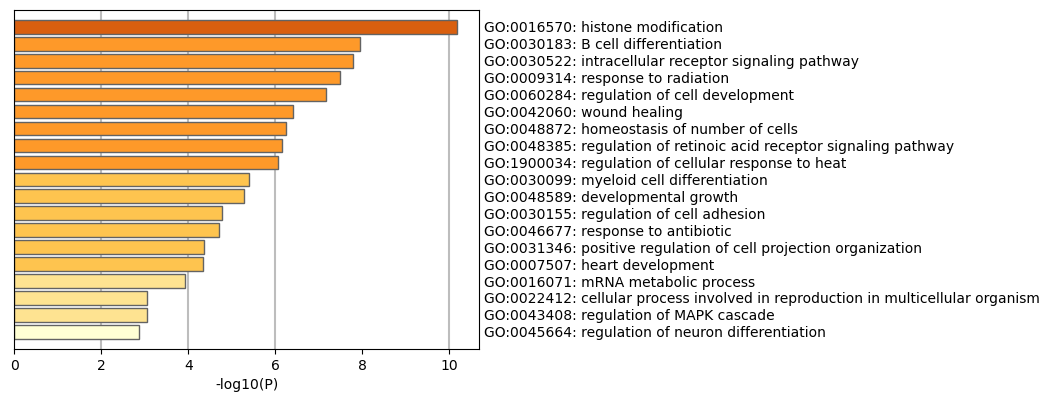
**

**B**

**
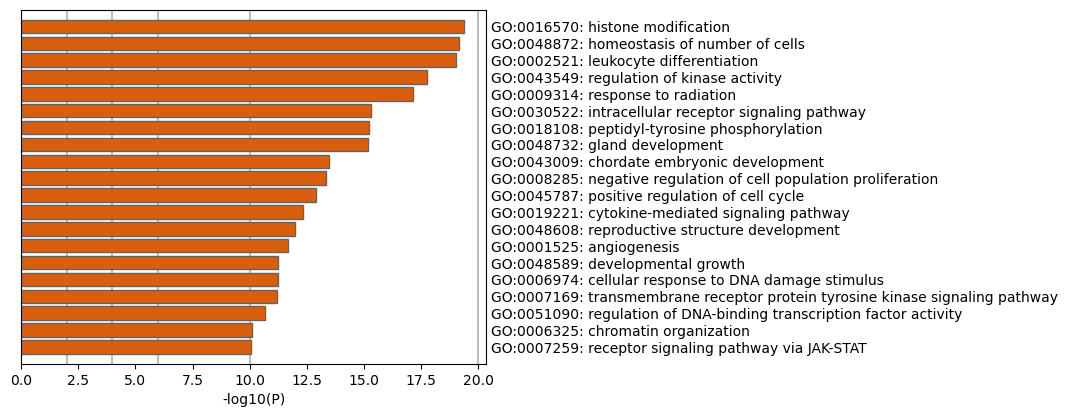
**

**C**

**
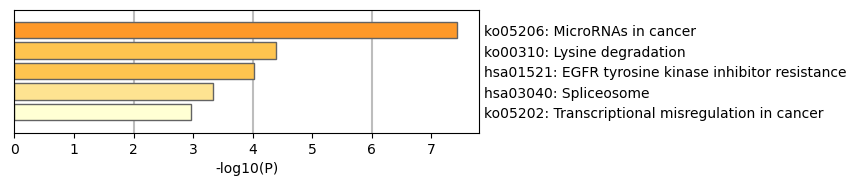
**

**D**

**
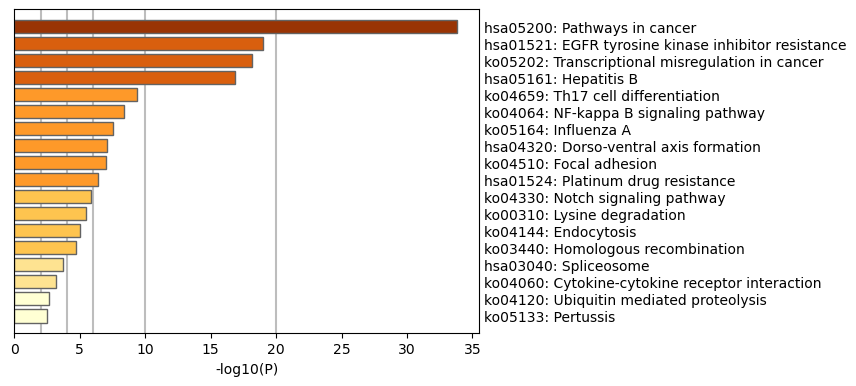
**

**E**


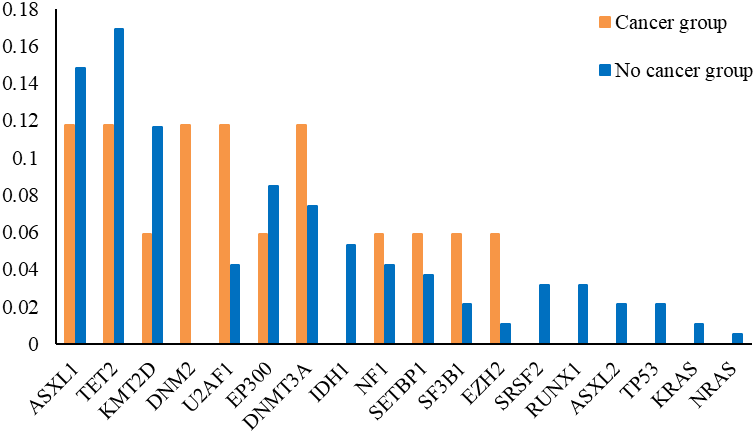


**Supplementary Figure 2. Next-generation sequencing analysis.** **A, GO functional enrichment analysis in the cancer group. B, GO functional enrichment analysis in the noncancer group. C, KEGG pathway enrichment analysis in the cancer group. D, KEGG pathway enrichment analysis in the noncancer group. E, Proportions of gene mutations in the cancer group and the no cancer group.**
